# Nutritional Value and Bioactive Compounds of Leaves and Grains from Quinoa (*Chenopodium quinoa* Willd.)

**DOI:** 10.3390/plants11020213

**Published:** 2022-01-14

**Authors:** Elena Villacrés, María Quelal, Susana Galarza, Diana Iza, Edmundo Silva

**Affiliations:** 1 Departamento de Nutrición y Calidad, Instituto Nacional de Investigaciones Agropecuarias, Mejía 171108, Ecuador; maria.quelal@iniap.gob.ec; 2 Facultad de Ciencias Agropecuarias y Recursos Naturales, Universidad Técnica de Cotopaxi, Latacunga 050108, Ecuador; susi29m@hotmail.com; 3 Facultad de Ciencias Químicas, Universidad Central del Ecuador, Quito 170129, Ecuador; diana.9122@outlook.com; 4 Facultad de Ingeniería Química, Universidad de Guayaquil, Guayaquil 090514, Ecuador; edmundosilvapaguay@hotmail.es

**Keywords:** quinoa grains, leaves, nutritional value, bioactive compounds, antinutritional factors

## Abstract

Quinoa is an important crop for food security and food sovereignty in Ecuador. In this study, we evaluated the nutritional value, bioactive compounds, and antinutrient compounds of leaves and grains of the Ecuadorian quinoa variety Tunkahuan, and we identified significant differences between the nutrient content in the leaves and grains. The quinoa leaves presented a higher protein content than the grains, as well as inorganic nutrients such as calcium, phosphorus, iron, and zinc. Both the grains and leaves had an appreciable phenolic content. In addition, the quinoa grains presented a higher content of the antinutrient saponin than the leaves, while the leaves contained more nitrates and oxalates than the grains. Thus, quinoa leaves and grains exhibit excellent potential for application in the food and pharmaceutical industries.

## 1. Introduction

Quinoa is an important and environmental stress-tolerant pseudocereal with high nutritional value and a globally promoted cultivar for human consumption and nutrition [1]. Quinoa plants, originating from the Andean mountains in South America, have a large scale of biological diversity [2]. However, after being abandoned in favor of old-world crops, it is now starting to be rediscovered by modern scientific approaches [1].

Quinoa is a complete food with high-nutritional value due mainly to its high content of good quality protein and are also called “the mother grain” [3]. The Ecuadorian varieties of quinoa contain more fat and protein than other Andean varieties. Quinoa’s superiority over other grains (maize, wheat, and barley, among others) results from its richer protein, lipid, and ash content. Albumins and globolins represent the major storage quinoa proteins. In addition, quinoa proteins are accepted as high quality [4], and the lysine content is higher than that of rice and wheat [5]. According to the Food and Agriculture Organization (FAO), the quinoa grain is a vegetal food that provides all essential amino acids and fulfills the dietary requirement established for human nutrition [6]. The Tunkahuan quinoa variety was obtained by selection of a population of germplasm collected in the Province of Carchi (Ecuador) in 1985. Then, it was officially released as a variety in 1992. This variety is adapted in areas between 2200 m and 3200 m, and its yield is from 1500 kg/ha to 3000 kg/ha. Other important morphological descriptors of this variety are leaf size (large), leaf shape (triangular), days of panning (70–110), and days to harvest (150–210). Among the nutritional components, there are the protein (15.73%), fat (6.11%), and carbohydrate (60.37%) content [7]. 

Moreover, the grain contains adequate levels of micronutrients, such as vitamins and minerals (calcium, phosphorus, potassium, copper, iron, and zinc). Some factors such as the differences in drying and storage conditions, harvest time and fertilization, soil structure, climate, and irrigation affect the macro and micronutrient contents [8]. Antinutritional factors such as saponins, phytic acid, tannins, and protease inhibitors have also been identified in quinoa and can have a negative effect on the performance and survival of monogastric animals when it is used as the primary dietary energy source. Although, the presence of saponins is not limited to seeds and can be found in other parts of the plant, whose function is to protect against the adverse conditions of the outside environment. Although saponins impart a bitter flavor, they have found application in the production of hormones and immunological adjuvants and as potent natural insecticides [1,9]. Significant amounts of other bioactive compounds such as phytosterols, squalene, and other phenolic compounds, including quercetin and kaempferol glycosides, benzoic acids, protocatechuic acid, and vanillic acid derivatives, are also present in quinoa seeds. The antioxidant activity has been associated with their high content of phenolic compounds [4,10]. 

Research on quinoa has primarily focused on the nutrient composition of seeds, protein quality, starch functionality, and incorporation into food products made with cereal flours [11]. There are a lot of studies of quinoa leaves. The leaves contain many important potential nutrition, content of proteins, carbohydrates, lipids and dietary fiber, minerals such as Fe, Zn, Na, and K, and vitamins (provitamin A, vitamin E). Consumption of nutrient-rich leafy green quinoa may prevent nutritional deficiency caused by Fe and Zn [12,13]. The quinoa leaves have a flavor similar to spinach and can be used in salads or cooked as a green vegetable. Their nutritional composition is similar to spinach; though, the protein from quinoa leaves contains more isoleucine and valine and slightly less methionine, cystine, phenylalanine, and tyrosine [13].

Moreover, bioactive compounds present a better nutritional profile than grains. Some studies have evaluated the nutraceutical potential of quinoa leaves in the context of bioaccessibility and bioavailability of their phenolic compounds, with in vitro studies suggesting that they can help prevent cancer and other diseases related to oxidative stress [14,15]. The aim of this study was to characterize the nutritional composition and bioactive compounds in the leaves and grains of the Tunkahuan variety of quinoa (*Chenopodium quinoa* Willd.), produced in Ecuador. 

## 2. Results

### 2.1. Proximate Composition 

Table 1 indicates the proximate composition and dietary fiber content of the quinoa leaves and grains. Statistical analysis (Student’s *t*-test) confirmed significant differences between the leaves and grains. The ash and protein contents of the leaves were higher than those of the grains. In addition, the lipid content (ether extract) in grains was higher than in quinoa leaves, maize, and wheat. The crude protein content in leaves was higher than quinoa grains. The crude fiber content in leaves and grains was higher than that in maize, wheat, and spinach leaves. The quinoa leaves contained more dietary fiber (soluble and insoluble) than the grains. 

### 2.2. Amino Acid Analysis

The amino acid profiles of quinoa grains and leaves are presented in Table 2. Statistical analysis (Student’s *t*-test) confirmed the significant differences between leaves and grains and other plants. Essential amino acids (EAAs) such as phenylalanine, lysine, isoleucine, and threonine were found in higher proportions in the quinoa leaves than in the grains. In addition, the leaves presented higher lysine and isoleucine levels than grains of maize and wheat [18]. Other non-essential amino acids (NEAAs), such as glutamic acid, aspartic acid, and alanine, were present in significantly higher amounts in leaves than in quinoa grains and maize, wheat, and spinach leaves. Thus, both the leaves and grains of quinoa exhibited a higher content of NEAAs than EAAs, with the EAA content approximately 35% more than that of NEAAs. In contrast, the EEA/NEAA ratios in wheat, maize, and spinach were higher than that in quinoa grains and leaves. 

### 2.3. Mineral Content 

The macro and microminerals analyzed are listed in Table 3. There were statistically significant differences in mineral content between the quinoa leaves and grains. The ash content of the leaves was higher than that of quinoa grains, spinach leaves, and other grains. The leaves also contained calcium, magnesium, and potassium, and the microelements iron and zinc were in considerably higher quantities compared to other minerals. The calcium content in quinoa grains was higher than that in maize and wheat. However, the phosphorus content was higher in wheat than in quinoa grains. The quinoa leaves presented larger concentrations of valuable macroelements than spinach leaves, and higher iron and zinc levels than those of maize and wheat. 

### 2.4. Antioxidant and Antinutritional Compounds

The compounds with antioxidant properties analyzed in this study are shown in Table 4. Statistical analysis (Student’s *t*-test) confirmed significant differences between leaves and grains in the content of antioxidant compounds, although the estimated antioxidant capacity was not significantly different. A high content of ascorbic acid was observed in the quinoa leaves, whereas it was not detected in the grains. Carotenoids, total phenols, flavonoids, and anthocyanins were also present in higher amounts in the quinoa leaves than in the grains. Among the antinutritional factors, saponin substances were present in higher concentrations in the grains than in the leaves, whereas nitrates and oxalates were more predominant in the leaves. 

## 3. Discussion

The proximate composition of quinoa leaves and grains has been demonstrated to be richer than that of other cereals and leaves. In another study, the crude protein content of quinoa leaves was reported to be 14.77 g/100 g (dry weight) [20]. Recent studies of crops of the same varieties (Bolivian and Argentinian) grown in different environments also reveal differences in total protein content, but without substantial losses and broadly maintaining the balance between the different amino acids [21,22]. In another study, with different quinoa varieties (Inia431-Altiplano, White, Titicaca, Illpa Inia, and Carmen) obtained from Adana Eastern Mediterranean Agricultural Research Institute, the protein content in the seed was within 12.96–16.84% [8]. The protein content of grains determined in this study was similar to values reported in other studies on different cultivars of quinoa [22,23,24]. The differences in protein content may be related to crop conditions; if the soil is rich in nitrogen, the plant produces more proteins than a plant cultivated in nitrogen-poor soil [8,13,23]. Nevertheless, the protein content of quinoa leaves (27.84 g/100 g as determined in this study) was higher than that from spinach (25.67 g/100 g), amaranth, and moringa leaves. Leaf protein represents an important nutritional component and could play a key role in alleviating nutritional deficiency in rural populations [13,17]. 

Grain oil is of high nutritional quality, containing several essential fatty acids; the predominant oleic and linoleic acids confer several positive health effects [1,25]. Both the leaves and grains analyzed in this study presented similar crude fiber content but were higher than that of lettuce (0.7 g/100 g) and spinach (0.6 g/100 g) [17]. Thus, quinoa leaves could be a good source of fiber that a normal body needs to function. 

The major components of dietary fiber include cell wall polysaccharides (galacturonic acid, arabinose, galactose, xylose, and glucose). Dietary fiber is divided into soluble and insoluble fractions based on water solubility [26]. In this study, the soluble and insoluble dietary fiber contents of leaves were higher than those grains. Together, the total dietary fiber content of quinoa leaves and grains was similar to that of cereals such as wheat [4]. Repo-Carrasco and Serna [21] determined the contents of dietary fiber in quinoa samples to be 11.99–14.39% (IDF) and 1.41–1.60% (SDF). For yellow quinoa of 25 different cultivars, the ranges of insoluble and soluble fiber contents were 10–14, and 3.7–5.9% respectively [27]; these results could be influenced by the quinoa variety, crop conditions, genetic conditions, and growing locations, which can all affect the fiber composition of quinoa [28,29]. The World Health Organization (WHO) recommends a dietary fiber intake of 20–35 g/per day for adults, in 3:1 proportion of IDF and SDF [26], which can be supplied by quinoa. Other studies have reported that the high fiber content of quinoa can improve digestibility by facilitating the absorption of other nutrients present in quinoa in the large intestine [9]. The quinoa fiber had more soluble fiber than cereals. The fiber composition was more similar to fruits than the cereals and had great potential to improve the functional and nutritional properties of food products [26].

Quinoa is one of the few plant foods that provides essential amino acids. Thus, quinoa proteins are accepted as high quality protein because of their balanced composition of essential amino acids, with the exception of tryptophan [4,5]. The quinoa grains contained higher amounts of valine and histidine than the leaves. Based on FAO/WHO/ United Nations (ONU)-recommended values, histidine, isoleucine, lysine, and valine amino acids can meet the requirements of school children and adults [16,19]. In addition, quinoa grains have higher lysine content than other cereals; some studies attributed the high lysine content to the existence of seven new proteins (globulins) similar to vicilin (legume reserve proteins) [30,31,32].

The macro and microminerals in quinoa leaves and grains were found at concentrations greater than those reported for maize, wheat, and spinach leaves. Several authors have reported that the mineral content of quinoa is adequate for a balanced diet [4]. Thus, the iron in quinoa shows good bioavailability [16], although antinutrients such as saponins and phytic acids can affect its bioavailability [33]. In addition, differences in mineral content have been observed in various studies on quinoa, which has been attributed to differences in plant genotypes, soil mineral composition or fertilizer application, uptake of minerals by individual plants, and organic soil amendments [9,13]. 

Bioactive compounds of quinoa were found mainly in the outer layers of the seeds, functioning as a chemical defense against insects and microorganisms [4,34]. Studies have demonstrated that antioxidant capacity in quinoa seeds is associated with the high content of phenolic compounds [4,35]. Quinoa leaves and seeds can provide important bioactive compounds with positive health effects. The total phenolic content of Tunkahuan leaves was significantly higher than that reported in other studies with other varieties of quinoa leaves [36,37]. In the seeds, the phenol content was similar to that of the red and white quinoa varieties derived from Bolivia and Chile [36]. For yellow quinoa of 25 different cultivars, the amount of phenolics in the cultivars were higher than reported in this study [27]. 

Some studies have established the chemical structure of phenolic compounds that confers potent antioxidant properties exerted through different mechanisms. They have the ability to scavenge free radicals and chelate metal ions, inhibit oxidases, terminate radical chain reactions, and stabilize free radicals [32]. 

In the case of flavonoids and anthocyanins, the leaves contained concentrations 98.47 times higher than those present in quinoa seeds. In another study, the flavonoid content of *Chenopodium* species varied from 36.2 to 144.3 mg/100 g, and the predominant flavonoids were shown to be quercetin and kaempferol [38]. The rutin flavonoid content in leaves of two quinoa cultivars were reported to be 869 mg and 914 mg/100 g [37]. The antioxidant capacity depends on the number and arrangement of hydroxyl groups in the food, which can decrease the progress of oxidation by transferring hydrogen atoms to radicals [39]. As shown in Table 4, the leaves had a slightly higher antioxidant capacity than the grains. The study has shown that the variation in polyphenol content among quinoa cultivars is linked to soil, climatic conditions, agronomical practices, genetic interactions (variety and cultivar), and stress conditions [40]. Thus, other non-phenolic compounds, such as ascorbic acid and carotenoids, may be the most likely contributors to the antioxidant activity in quinoa [9]. Finally, most reports on the nutraceutical potential of quinoa focus only on seeds, whereas only little information is available on the antioxidant potential of quinoa leaves [36]. Our results indicate that the leaves could offer important health benefits. 

With respect to antinutritional compounds, other researchers have reported saponin content of 1260 mg/100 g and 970 mg/100 g in quinoa seeds and leaves, respectively [41]. The saponin content of quinoa varieties grown in Washington State, USA ranged from 381 mg/100 g to 2710 mg/100 g [42]. Washing the grains under running water and scrubbing can reduce the saponin content, although it also appears to affect the mineral content; the different analytical methods and the different levels of irrigation and water salinity could also affect the saponins content within the same cultivar. Some studies suggested that the saponins content increases with higher levels of water salinity and decreases with lower irrigation levels [41,43]. In contrast to saponin, nitrates and oxalates are present at higher concentrations in leaves than in seeds. Nitrates are concentrated in vacuole leaves and transport organs but are less abundant in flowers, tubers, and seeds [44]. The nitrate concentration in quinoa leaves is lower than that in amaranth and sangorache leaves (122.83 mg/100 g and 260 mg/100 g, respectively), and in the seeds, it reaches concentrations of 20.01 mg/100 g (amaranth) and 34.61 mg/100 g (sangorache) [45]. Nitrogen fertilization and light intensity have been shown to be a major influence on the nitrate content of vegetables [44].

The concentration of oxalates in the leaves was 66.46% higher than that in the quinoa grain. In another study, the oxalate concentration in amaranth and quinoa grains were 184 mg/100 g to 232 mg/100 g dry weight, respectively [46]. However, a high dietary oxalate intake can influence mineral and trace element absorption in humans and may lead to calcium oxalate stone formation due to the ability of oxalate to form insoluble complexes with divalent cations in the gastrointestinal tract [46]. Thermal processes (boiling, blanching) and soaking can contribute to reducing the content of antinutrient compounds, as these compounds are highly water-soluble [45,46].

## 4. Materials and Methods

### 4.1. Plant Material 

Iniap Tunkahuan (Ecuador) quinoa leaves and grains were selected for this study. The plants were provided by the National Legumes Program and INIAP Andean Grains (Ecuador). They were grown in the Santa Catalina Experimental Station with the geographic location of altitude 3050 m.a.s.l, latitude UTM 9959382 m S, longitude 17 M0772618 m W. Ecuador does not have a defined season, but the quinoa was grown with an average rainfall of 600 mm, temperature variation (7–17 °C), and sandy loam soil (pH 6.5). In the case of the soil, the fertilization application was 80 kg of nitrogen and 40 kg of P_2_O_5_ per hectare were applied for fertilization.

The quinoa leaves were harvested manually after cultivation for 80 days. The grains were harvested with a reaper for 170 days, threshed and classified in a Crippen Mfg. Inc (St. Louis, MO, USA) using sieves with pore sizes of 2.5 mm, 2.2 mm, and 2 mm. For this study, grains with an average diameter of 2.5 mm were used.

The samples were taken to the laboratory, classified, placed in polypropylene bags, frozen at −20 °C for 24 h, and then freeze-dried using LABCONCO equipment (Kansas, MO, USA). The seeds were cleaned of dust and other contaminants. The leaves and grains were milled (Retsch KG 5657 Haan, Remscheid, Germany) to a particle size of 250 µm, and the ground powder was packed in polypropylene bags and stored in a cool, dry place (4 °C) until further analysis. 

### 4.2. Proximate Compositon 

The protein, fat, crude fiber, and ash contents were determined according to the Association of Official Analytical Chemistry (AOAC) standardized methods [47]. Carbohydrate content was calculated using the difference in dry weight. 

### 4.3. Soluble and Insoluble Dietary Fiber

SDF and IDF were quantified by sequential enzymatic digestion of quinoa leaves and grains by heat-stable α-amylase, protease, and amyloglucosidase. The IDF was filtered, and the residue was washed with distilled water, ethanol, and acetone. The combined solution of filtrate and water washings were precipitated with ethanol for SDF determination. The precipitate was filtered and dried, and both residues (IDF and SDF) were corrected for protein, ash, and blank for calculation of fiber content (AOAC 991.43) [48].

### 4.4. Amino Acid Analysis 

The amino acid profile was determined using high-performance liquid chromatography. The processed leaf and grain samples were applied to an LC-10AS Shimadzu liquid chromatography system, which operated under the following conditions: oven temperature, 60 °C; excitation wavelength, 350 nm; emission wavelength, 450 nm; sample cooler temperature, 4 °C; eluent solution flow, 0.60 m^2^ min^−1^; injection volume, 5 μL; run time, 45 min [47].

### 4.5. Mineral Composition 

The mineral content in quinoa leaves and grains was determined using wet oxidation and atomic absorption spectrophotometry on an AA-7000 spectrophotometer (Shimadzu, Kyoto, Japan), except for phosphorus, which was analyzed by colorimetry [47]. 

### 4.6. Antioxidant Compounds 

#### 4.6.1. Total Carotenoids 

Total carotenoid content was determined using cold acetone and petroleum ether. Absorbance was measured at 450 nm. The carotenoid content was calculated using an extinction coefficient of 2500 [49]. 

#### 4.6.2. Ascorbic Acid 

Ascorbic acid was determined by reflectometric analysis using a test strip that monitors the reduction of yellow molybdophosphoric acid to phosphomolybdenum blue by ascorbic acid. The processed quinoa samples were diluted in distilled water and stirred thoroughly. The test strip was then dipped in the solution, excess liquid was allowed to run off via the long edge of the strip on to absorbent paper, and then the strip was analyzed using a reflectometer. The results were expressed in mg/100 g dry weight [50]. 

#### 4.6.3. Polyphenolic Compounds 

Polyphenolic compounds were quantified using Folin Ciocalteau 2N reagent; the absorbance was measured at 765 nm, and the results were expressed in mg gallic acid/100 g dry sample [51]. Total anthocyanins were extracted with ethanol solution and HCl and were quantified by measuring absorbance at 515 nm on an Evolution 201 spectrophotometer (Thermo Fisher Scientific, Waltham, MA, USA). The total anthocyanin content was calculated using an extinction coefficient of 27300 [52]. 

#### 4.6.4. Antioxidant Capacity 

Trolox equivalent antioxidant capacity (TEAC) was determined by measuring reduction of 2,2′-azino-bis (3-ethylbenzothiazoline-6-sulfonic acid) in 80% methanol and comparing against a Trolox standard curve. TEAC was expressed as µmol Trolox equivalent/g dry weight [53]. 

### 4.7. Antinutritional Compounds 

#### 4.7.1. Nitrates 

Nitrate content was determined using a test strip that measures the reduction of nitrate ions by a reducing agent. The processed quinoa leaf samples were added to distilled water and were homogenized and centrifuged for 10 min at 10,000× *g*. Then, sulfuric acid (72%) and distilled water were added to a portion of the extract and were incubated for 2 h at 100 °C. The strip with the sample was analyzed using a reflectometer. The results were expressed as mg/100 g (dry weight basis) [54]. 

#### 4.7.2. Saponin Content

Saponin content was determined by spectrophotometry. Samples were first homogenized with ethanol (25%); the extract was filtered, and then an aliquot of the extract was mixed with a color reagent (sulfuric acid:acetic anhydrous 3:1 *v*/*v*). Absorbance was measured at 528 nm and compared against a pure saponin standard. The saponin content was expressed as mg/100 g dry weight [55]. 

#### 4.7.3. Oxalic Acid 

Oxalic acid concentration was determined by titration with 0.001 N KMNO_4_ according to a previously reported method, with some modifications. The samples were placed in a muffle furnace for 10 h at 350 °C. H_2_SO_4_ and distilled water (25 mL) were then added to the ash. The solution was filtered, warmed to approximately 60 °C, and used for titration. An oxalic acid equivalent (45 Eq/g) was used for the calculation [56]. 

### 4.8. Statistical Analysis 

The samples were analyzed in triplicate, and the results are expressed as mean and standard deviation (SD). Student’s *t*-test was used to identify significant differences between leaves and grains. The INFOSTAT software package was used for the statistical analysis [57]. 

## 5. Conclusions

Quinoa is a pseudocereal with high nutritional value. The leaves and grains of quinoa could be considered functional foods as they contain adequate protein, dietary fiber, minerals, and important bioactive compounds. However, quinoa can also present antinutrient compounds, whose content can vary depending on the plant organ, and can be reduced using thermal processing. Moreover, the leaves have a higher mineral content and may help to overcome nutritional deficiencies. Antioxidant compounds such as ascorbic acid, carotenoids, and phenolic compounds are also highlighted. The leaves have antinutritional compounds; however, the levels found in the leaves are low and within what is allowed for human consumption. The results of the research show that the leaves can be used in culinary preparations, in the elaboration of functional foods, and in the extraction of bioactive compounds for the food or pharmaceutical industry.

## Figures and Tables

**Table 1:** Comparison of proximate composition and dietary fiber content of quinoa leaves with quinoa grains and other plant species (g/100 g dry weight of the sample).

Parameters	Leaves	Quinoa Grains	Maize ^a^	Wheat ^a^	Spinach Leaves ^b^
Moisture	5.23 ± 0.17	12.00 ± 0.11 **			
Ash	19.86 ± 0.16	1.96 ± 0.15 **	1.7 **	2.2 **	13.45 *
Ether extract	3.50 ± 0.21	6.00 ± 0.22 **	4.70 *	2.3 *	4.65 *
Protein	27.84 ± 0.36	16.70 ± 0.30 **	10.2 **	14.3 **	25.67 *
Crude fiber	8.02 ± 0.24	8.61 ± 0.21 *	2.3 **	2.8 **	7.33 *
Nitrogen free extract	40.78 ± 0.40	66.73 ± 0.45 **	81.1**	78.4 **	48.90 *
Soluble dietary fiber (SDF)	3.29 ± 1.17	0.74 ± 0.28 **			
Insoluble dietary fiber (IDF)	33.07 ± 1.17	10.36 ± 0.14 **			
Total dietary fiber	36.37 ± 2.34	11.10 ± 0.15 **			

Mean ± standard deviation (n = 3); ** highly significant; * significant; ^a^ Data according Koziol [16]; ^b^ Data according Hanif et al. [17].

**Table 2:** Comparison of amino acid profiles of quinoa leaves with quinoa grains and other plants (g/100 g protein dry weight of the sample).

Amino Acids	Leaves	Quinoa Grains	Maize ^a^	Wheat ^a^	Spinach Leaves ^b^	School Children ^+^
**EAAs:**						
Histidine	1.90 ± 0.17	2.31 ± 0.25 *	2.6 *	2.0	2.5 *	1.9
Isoleucine	4.13 ± 0.13	2.87 ± 0.13 *	4.0	4.2	4.8	2.8 *
Lysine	4.24 ± 0.16	3.81 ± 0.29 *	2.9 *	2.6 *	7.3 **	4.4
Threonine	3.88 ± 0.10	2.62 ± 0.17 *	3.8	2.8 *	5.3 *	2.8 *
Valine	2.84 ± 0.14	3.81 ± 0.20 *	5.0 *	4.4 *	6.1 *	2.5
**NEAAS:**						
Tyrosine	2.91 ± 0.04	2.19 ± 0.10	0.7 *	0.2 **	ND	0.9 **
Phenylalanine + tyrosine	5.42 ± 0.36	3.31 ± 0.23 *	8.6 **	8.2 **	11.1 **	2.2 *
Glutamic Acid	16.12 ± 0.22	13.44 ± 0.40 *	18.8 *	29.5 **	11.7 *	
Aspartic Acid	10.69 ± 0.28	7.68 ± 0.35 *	6.9 *	5.0 *	9.9	
Alanine	10.41 ± 0.39	2.87 ± 0.35 **	7.3 *	3.6 **	6.3 *	
Glycine	8.90 ± 0.12	11.00 ± 0.40 *	4.0 **	4.0 **	5.2 **	
Arginine	5.06 ± 0.08	6.50 ± 0.16 *	4.2 *	4.5 *	6.4 *	
Proline	4.49 ± 0.39	2.87 ± 0.12 *	9.1 **	10.2 **	4.8	
Serine	4.05 ± 0.06	8.19 ± 0.10 *	5.1 *	4.8	4.8	
**Nutritional evaluation**						
EAA	22.4	18.7 *	26.90 *	24.20 *	37.10 **	
NEAA	63.3	55.0 *	56.10 *	61.80 *	49.10 **	
TAA	85.7	73.8 *	83.00 *	86.00	86.20	
EAA/TAA (%)	26.2	25.4	32.41 *	28.14 *	43.04 **	
NEAA/TAA (%)	73.8	74.6	67.59 *	71.86	56.96 **	
EEA/NEAA (%)	35.4	34.0	47.95 **	39.16 *	75.56 **	

Mean ± standard deviation (n = 3), EAA = essential amino acid; NEAA = non-essential amino acid; TAA = total amino acid; ND = not detectable. ** highly significant; * significant; ^a^ Data according Koziol (1992) [16], ^b^ Data according Hanif et al. (2006) [17], ^+^ Data according Values from FAO (1985) [19].

**Table 3:** Comparison of macro and microminerals in quinoa leaves with quinoa grains and other plants (dry weight of the sample).

Minerals	Leaves	QuinoaGrain	Maize ^a^	Wheat ^a^	Spinach Leaves ^b^
Macroelements ^1^					
Calcium	2.79 ± 0.28	0.18 ± 0.03 **	0.02 **	0.05 **	0.08 **
Phosphorus	0.63 ± 0.25	0.32 ± 0.07 *	0.29 *	0.47 *	0.08 **
Magnesium	2.26 ± 0.23	0.16 ± 0.08 **	0.14 **	0.17 **	NR
Potassium	4.74 ± 0.21	0.33 ± 0.12 **	0.34 **	0.58 **	0.20 **
Sodium	0.05 ± 0.01	0.02 ± 0.12 *	0.01 *	0.01 *	0.06 *
Microelements ^2^					
Copper	8.00 ± 0.50	0.60 ± 0.10 **	NR	7 *	0.000005 **
Iron	483.00 ± 1.00	7.80 ± 1.31 **	21 **	38 **	0.006 **
Manganese	38.00 ± 1.00	1.00 ± 0.50 **	5 *	39	NR
Zinc	204.00 ± 2.00	3.30 ± 0.52 **	29 **	47 **	NR

Mean ± standard deviation (n = 3). ^1^ Expressed as g/100 g. ^2^ expressed as mg/kg. NR: not reported; ** highly significant; * significant; ^a^ Data according Koziol (1992); ^b^ Data according Hanif et al. (2006) [17].

**Table 4:** Antioxidant and antinutrient compounds in quinoa leaves and grains (dry weight of the sample).

Antioxidant Compounds	Leaves	Grain
Ascorbic acid (mg/100 g)	358.00 ± 0.17	ND
Total carotenoids (µg RE/g)	1695.12 ± 5.22	13.89 ± 1.01 **
Total phenols (mg AGE/100 g)	4902.53 ± 6.4	1110.39 ± 1.63 **
Flavonoids (mg quercetin/100 g)	616.00 ± 0.268	9.48 ± 1.12 **
Anthocyanins (mg/100 g)	49.36 ± 0.56	0.77 ± 0.21 **
Antioxidant capacity (µMoles Trolox equivalent/g)	44.16 ± 0.72	31.99 ± 1.79
**Antinutrient compounds**		
Saponins (mg/100 g)	0.49 ± 0.01	199.07 ± 1.01 **
Nitrates (mg/100 g)	60.07± 1.14	21.83 ± 1.61 **
Oxalates (mg/100 g)	12.85 ± 0.25	4.31 ± 0.47 **

Mean ± standard deviation (n = 3). RE: retinol equivalents. AGE: gallic acid equivalent. ND: not detectable. ** highly significant.

## Data Availability

The data presented in this study are available on request from the corresponding author.
